# Impact of routine use of the GFAP and UCH-L1 TBI blood test for management of mild TBI on patient care in a European emergency department

**DOI:** 10.1007/s00068-026-03303-4

**Published:** 2026-08-14

**Authors:** Felix Brüning-Wolter, Nicola Wolff, Meike Schrader, Ksenia Musaelyan, Raj Chandran, Markus Hoffmann, Jaime A. Marino, Beth McQuiston, Jörg Cramer

**Affiliations:** 1 Department of Trauma Surgery, Orthopaedics and Hand Surgery, Marienkrankenhaus, Hamburg, Germany; 2 Emergency Department, Städtisches Klinikum Lüneburg, Lüneburg, Germany; 3 Laboratory Medicine Department, Städtisches Klinikum Lüneburg, Lüneburg, Germany; 4 Abbott Core Diagnostics, Abbott Park, IL USA; 5 Orthopedics and Traumatology Department, Städtisches Klinikum Lüneburg, Lüneburg, Germany

**Keywords:** Traumatic brain injury, Biomarkers, GFAP, UCH-L1

## Abstract

**Objectives:**

Blood biomarkers glial fibrillary acidic protein (GFAP) and ubiquitin C-terminal hydrolase L1 (UCH-L1) were recently introduced into clinical practice for mild traumatic brain injury (mTBI) assessment. However, real world data demonstrating TBI biomarkers’ impact on the emergency department (ED) practice is lacking. This study aimed to describe the experience of a single tertiary hospital ED that introduced TBI biomarkers GFAP and UCH-L1 measurements using Alinity i^®^ TBI test into routine mTBI assessment.

**Methods:**

Retrospective data were extracted from hospital records of patients who had TBI test ordered in the ED. Test results and subsequent clinical care pathway data (CT order, CT scan result, admission or discharge) were analysed.

**Results:**

Data was available for 460 mTBI patients (median age 43, 54% male). Of the 216 patients (47%) with a negative TBI test, 198 did not have a CT scan, and 18 had a CT scan with no abnormalities detected. Among 244 patients with a positive TBI test, 162 had CT scans, with 12 (6.6%) showing intracranial lesions. Overall, among all 180 scanned patients every patient with intracranial lesion findings had a positive TBI test result. The test could rule out CT scans in 47% of mild TBI patients. In practice, 42% of patients avoided a CT scan based on the TBI test results with additional 12% of patients ruled out based on clinical assessment.

**Conclusions:**

Routine TBI test use spared over 40% of patients from CT scanning, with further potential to reduce unnecessary imaging through improved protocol adherence.

## Introduction

Traumatic brain injury (TBI) is one of the most common neurological conditions seen in the emergency room and presents a significant burden on the healthcare system and society [1]. After an initial clinical examination and determination of the Glasgow Coma Scale (GCS), 80–90% of TBI patients are categorized as mild [2]. Standardised assessment and care for these patients remains a challenge in the emergency department (ED), as diagnostic criteria for mild TBI (mTBI) are not well-defined and often vary not just between countries but even between individual hospitals [3, 4].

According to most currently used guidelines, CT scan remains a gold standard of mTBI assessment to avoid life-threatening complications caused by rare intracranial haemorrhages requiring urgent neurosurgical intervention. However, most of the CT scans conducted for mild TBI patients (up to 95%) do not show any abnormalities [5, 6], which signifies suboptimal use of this diagnostic modality. Such excessive practice is especially problematic in view of ever-increasing pressures of waiting times at the ED and in the radiology department, as well as in relation to increased cancer risk associated with CT scan-associated dose of X-irradiation exposure. Recently, several national and international consensus recommendations incorporated the use of blood biomarkers glial fibrillary acidic protein (GFAP), ubiquitin C-terminal hydrolase L1 (UCH-L1) and S100b to rule out patients at low risk of having any intracranial lesions visible on a CT scan [7–10]. GFAP is an intermediate filament protein highly specific to astrocytic cytoskeleton, with no significant expression described outside of the nervous tissue [11]. UCH-L1 is a neuronal isoform of the ubiquitin degradation cascade enzyme present in neuronal cell bodies with some extracranial expression described in gonadal tissue and some cancers [12]. Both proteins are elevated in the blood of patients with intracranial lesions within the acute timeframe following TBI, thus making them suitable for TBI evaluation in the ED [13]. These biomarkers were recently introduced into the clinical routine use for the first time for mTBI management. Studies evaluating diagnostic performance of the novel TBI test panel (GFAP and UCH-L1) towards intracranial lesion rule out demonstrated high sensitivity (96.7%-100%) and negative predictive value (99.4%-100%) [14–17]. The advantage of the TBI test panel over previously introduced S100b is its ability to be used in patients seen within a broader time window of presentation at the ED (within 12 h from trauma), as well as its higher specificity towards brain injury [18, 19].

To improve and optimise clinical care for mTBI patients, a new clinical pathway was introduced at a single tertiary hospital which included the use of the TBI test panel to rule out patients at low risk of intracranial lesions seen on the CT scan. According to the new pathway, the test is indicated for all adult (aged 18+) mild TBI patients with GCS 13–15 who are seen within 12 h of trauma and are categorised as low or minimal risk. If both biomarkers GFAP and UCH-L1 are below their respective manufacturer developed cutoffs then the test is considered negative and in conjunction with other clinical information, a CT scan of the head can be ruled out. This study aimed to investigate the impact of introduction of the novel care pathway on routine TBI care in a single ED.

## Materials and methods

**Fig. 1:**
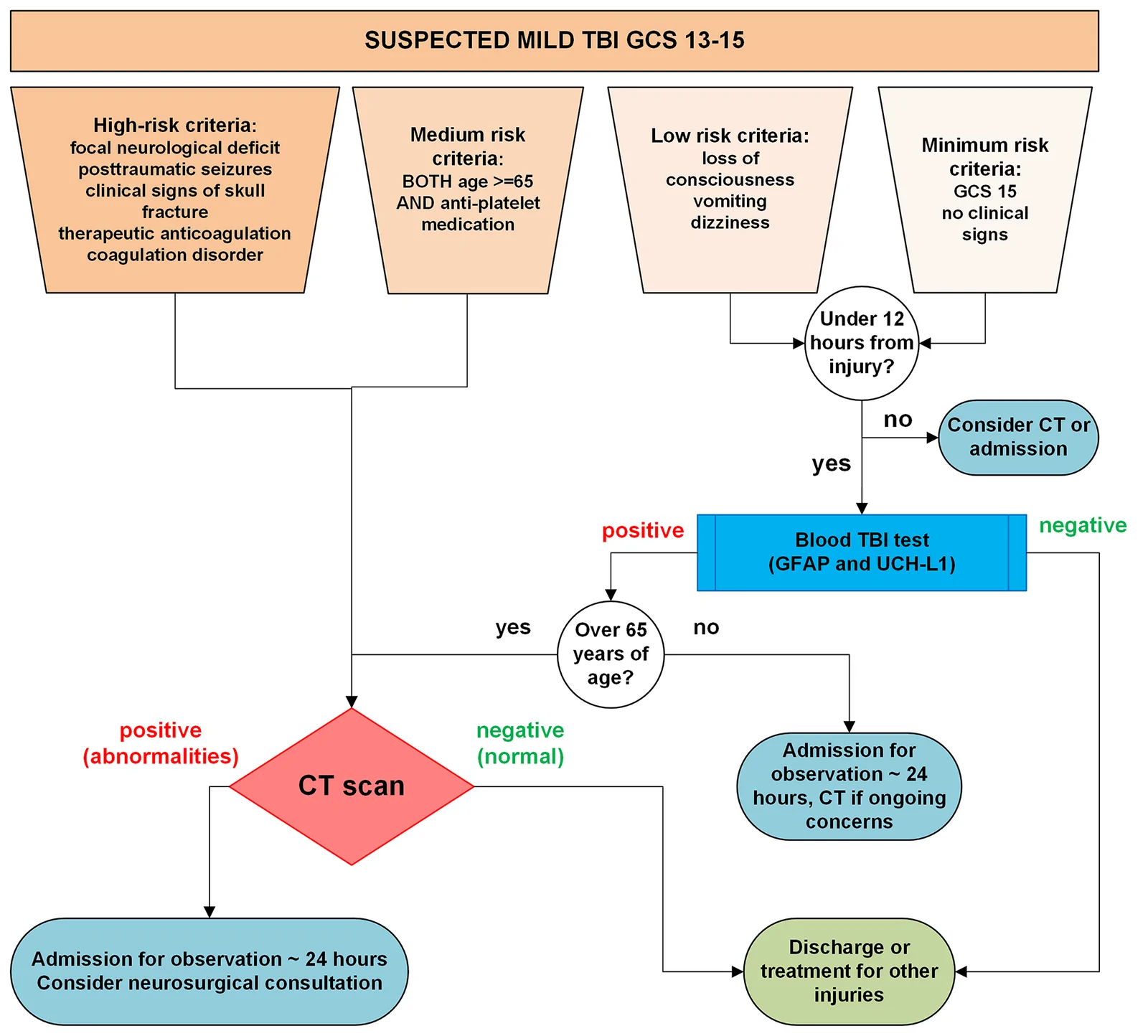
Clinical care pathway incorporating blood TBI test introduced at the ED at the time of test implementation. GCS – Glasgow Coma Scale, CT – computed tomography

Data was retrospectively extracted from medical records of patients who were seen in the Emergency Department of Städtisches Klinikum Lüneburg between May 2022 and December 2023 for suspected head injury and had a TBI test ordered by the attending team. Klinikum Lüneburg is a regional trauma centre serving a community of 150,000 people. Ethical approval of the retrospective analysis was granted by the Ethics Committee of the Medical Association of Lower Saxony (reference number Bo/08/2025-Studie-01, date of approval 20.03.2025). Patient selection criteria as part of the TBI management protocol introduced in the ED are shown in Fig. 1. The sample size was calculated targeting the number of subjects that can potentially be ruled out for head CT. Statistical assumptions were taken from the Abbott Alinity i TBI Test Package Insert [20]. Using an expected CT rule-out percent of 41% with an acceptable confidence width of ± 5% and CT positive prevalence of 6.2% it was found that a minimum of 409 subjects is needed using the Wilson Score sample size calculation method.

Extracted data included demographics, clinical assessment data, radiology data if radiological examination was ordered, and ED outcome (discharged/hospitalised for TBI/ treated for other injury/discharged against medical advice).

Blood was collected within 12 h from trauma. Venous blood samples were collected into S-Monovette^®^ Serum CAT tubes (SARSTEDT AG & Co. KG, Nümbrecht, Germany) and sent to the laboratory STAT. Blood samples were centrifuged at 3300–4000 rpm for 10 min. Samples were analysed fresh using automated Alinity i^®^ TBI test (Abbott Laboratories Inc., Sligo, Ireland) according to manufacturer’s instructions [20]. Briefly, Alinity i^®^ TBI test is a panel of in vitro diagnostic chemiluminescent microparticle immunoassays (CMIA) used for the quantitative measurements of glial fibrillary acidic protein (GFAP) and ubiquitin carboxyl-terminal hydrolase L1 (UCH-L1) in human plasma and serum and provides a semi-quantitative interpretation of test results derived from these measurements using the Alinity i^®^ System. 300 µl of plasma sample was loaded into the Alinity i^®^ system for analysis. Quantitative and automated qualitative results (positive or negative) were available on the system in 18 min from sample loading. The reportable range for GFAP is 3.2 pg/ml to 42,000 pg/ml. reportable range for UCH-L1 is 18.3 pg/ml to 25,000 pg/ml. Manufacturer provided cut offs were used for test interpretation. The test was interpreted as negative if GFAP value was below 35 pg/ml and UCH-L1 value was below 400 pg/ml. If one or both biomarker values were equal or above the respective cut offs the TBI test was interpreted as positive. Results were available to the attending team to aid in clinical decision making.

Acute pathological findings on the CT scan were used as a primary outcome. CT positive for intracranial lesions included findings such as epidural, subdural and subarachnoid haematomas, intraparenchymal contusions and bleeding, and any combination thereof.

Descriptive statistics was calculated using GraphPad Prizm 9.4.0. Rule out rates and diagnostic performance were calculated using Analyse-it version 5.90 (Analyse-it Software, Ltd (2021)) for Microsoft^®^ Excel^®^ for Microsoft 365 MSO (Version 2508). Confidence intervals for diagnostic parameters were calculated with Wilson Score method using SAS software version 9.4, SAS Institute Inc., 2023.

## Results

Data was available for 460 adult mild TBI patients seen within the initial 15 months of the TBI test introduction at Klinikum Lüneburg for whom Alinity i^®^ TBI test was ordered by the treating ED team. Clinical decision pathway utilised by the ED team for mTBI assessment is shown in Fig. 1. TBI test was indicated for mTBI patients (GCS 13–15) of low or minimal risk. Retrospective data analysis showed that TBI test was also applied to 3 patients with signs of neurological deficit and 37 patients aged 65 and older who were receiving anticoagulant medications.

Demographic characteristics of the cohort are shown in Table 1. The median age of patients was 43 years old, 54% of patients were male. 8 patients (1.8%) had a GCS score of 13. 35 patients reported or had a witnessed loss of consciousness (7.6%), 39 (8.4%) had post-traumatic amnesia. For information on other symptoms see Table 1. 84 patients (18%) were admitted into hospital with a TBI diagnosis following ED assessment. No deaths or neurosurgical interventions were reported.

**Table 1:** Demographic characteristics of the patient cohort

Characteristics	*N* = 460
Median Age (years)[min-max]	43 [18–97]
Male sex n(%)	249 (54.1)
Antithrombotic therapy n(%)	45 (12.1)
aspirin	21
apixaban	16
rivaroxaban	2
phenprocoumon	4
edoxaban	2
Alcohol intoxication n(%)	33 (7.1)
GCS Score	
13	8 (1.7%)
14	50 (11%)
15	402 (87%)
Symptoms	
LOC	35 (7.6%)
PTA	39 (8.4%)
Vomiting	15 (3.2%)
Dizziness	95 (20%)
Headache	252 (54.7%)
Neurological deficit	3 (0.6%)
No symptoms	91 (19.7%)
TBI Test positive n(%)	244 (53)
CT performed n(%)	180 (39)
CT findings n(%)	12 (6.6)
Subdural haematoma	4
Subarachnoid haematoma	3
Intraparenchymal bleeding	2
Combined lesions	2
Cerebral infarction	1
Outcome	
Discharged	308 (67%)
Admitted for TBI	84 (18%)
Treated for other injury	25 (5.4%)
Discharged against medical advice	43 (9.3%)

GCS – Glasgow Coma Scale; LOC – loss of consciousness; PTA – post-traumatic amnesia; CT- computed tomography; TBI – traumatic brain injury

**Fig. 2:**
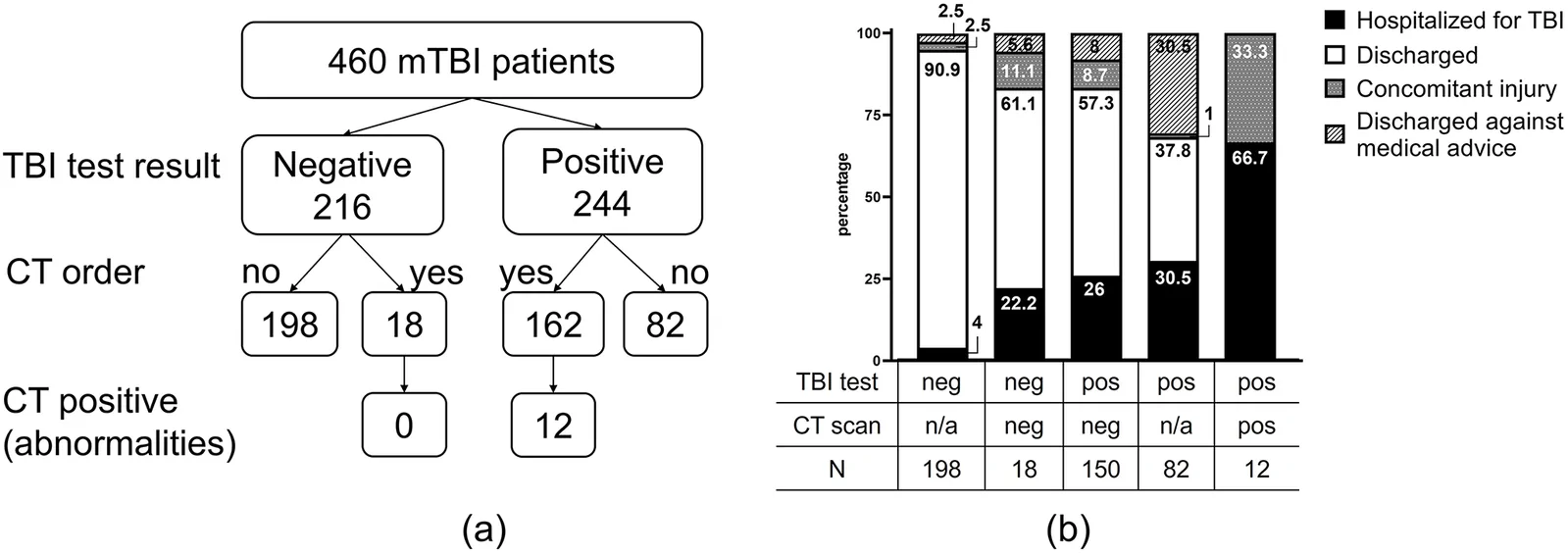
Patient outcomes (**a**) Patient care flow (**b**) ED assessment outcomes by TBI test and CT scan results groups. All data presented as percentages within each group. Neg – negative result; pos - positive result; n/a – CT scan not done; CT – computed tomography

Patient care flow is shown in Fig. 2 (a). Among 460 patients for whom TBI test was ordered, 216 (47%) had a negative TBI test result. The final decision regarding CT scan ordering rested with the treating physician. Among those with a negative TBI test result, 198 did not have a CT scan. Subsequently, 184 were discharged and 9 were admitted for TBI. 18 patients had a CT scan ordered despite a negative TBI test result – none of them had any abnormalities reported in the radiology report. 11 were discharged and 4 were admitted for TBI. For full outcome data see Table 2; Fig. 2 (b).

Among 244 patients with a positive TBI test result, 162 had CT scan ordered. Among those, 12 (6.6%) had intracranial lesion findings on the radiology reports, for detailed results see Fig. 2(b). All these patients were admitted for TBI or other injuries. Since detailed medical history was not available, one patient with findings consistent with ischemic stroke was excluded from the average biomarker level calculations (see Table 4).

82 patients had a positive TBI test but did not have a CT scan ordered. Among those 73 were below or at the age of 65 and thus were indicated to be admitted for observation as per care pathway in Fig. 1. Among the remaining 9 patients who should have had a scan according to the clinical pathway, 3 self-discharged against medical advice and 3 did not report any symptoms on admission. As the data was collected from hospital records retrospectively it is not possible to discern the exact reasoning behind clinician’s decision not to order the CT scan. However, it is interesting to note that among those 6 patients above 65 years old for whom the scan was not ordered against the clinical pathway recommendations GFAP levels measured below 80 pg/ml and all UCH-L1 levels measured below test positivity cutoff, with the highest value reported to be 303 pg/ml. Regarding other outcomes, 31 of the 82 non-scanned patients were discharged and 25 were admitted for TBI. For full outcome data see Table 2; Fig. 2 (b).

**Table 2:** Outcomes of the ED assessment of 460 suspected mTBI patients arranged by TBI test and CT scan results, n

TBI test result	Negative		Positive			Total
**CT scan**	**Not** **ordered**	**Negative (normal)**	**Not** **ordered**	**Negative (normal)**	**Positive (traumatic lesions)**	
Admitted for TBI	8	4	25	39	8	**84**
Discharged	180	11	31	86	0	**308**
Concomitant injury	5	2	1	13	4	**25**
Discharged against medical advice	5	1	25	12	0	**43**
**TOTAL**	**198**	**18**	**82**	**150**	**12**	**460**

CT – computed tomography, TBI – traumatic brain injury

Since the test was used clinically to rule out the need for a CT scan, outcome data (CT scan results) is not available for most of the test negative patients. Hence conventional calculation of test diagnostic performance was not possible in this real-world test use dataset. Based on CT scan results available for 180 patients, sensitivity of the TBI test was calculated to be 100% (CI 75.8–100) and negative predictive value was also 100% (CI 85.6–100). In the overall cohort of 460 patients TBI test results could allow to rule out CT scans in 47% of mild TBI patients. In practice, 54% of patients had CT scans ruled out by clinicians decision, 42% of patients supported by a negative TBI test, and 12% based on clinical judgement, see Table 3. 6.5% of patients self-discharged against medical advice without a scan. Overall, 280 patients (60.1%) did not have a CT scan (see Tables 2 and 3).

**Table 3:** TBI test and CT scan results in the patient cohort and intracranial lesion rule out rates based on available CT scan data, % from total cohort of 460 patients

CT scan decision		TBI test negative	TBI test positive	TOTAL
CT scan ordered	CT negative, *n* (%)	18 (4%)	150 (33%)	168
	CT positive, *n* (%)	0	12 (3%)	12
CT scan not ordered	By clinician’s decision, n (%)	193 (42%)	57 (12%)	250
	Due to patient self-discharge, n (%)	5 (1%)	25 (5.5%)	30
**Total**		**216 (47%)**	**244**	**460**

CT – computed tomography, TBI – traumatic brain injury;

Outcomes for all groups of patients are shown in Table 2; Fig. 2 (b). Of note, 26% of TBI test negative and CT negative patients, as well as 25% of TBI positive CT negative patients were admitted for further observation or treatment of TBI. 31% of patients with positive TBI test result and without a CT scan done were admitted into hospital for further observation or treatment for TBI. 32.5% of these patients were discharged against medical advice. All patients with abnormalities on the CT scan were further admitted either for TBI or other injuries.

Since TBI test provides numerical values for each of the two biomarkers, it was possible to calculate median biomarker values per each patient group, see Table 4.

**Table 4:** Median biomarker levels by TBI test and CT scan result groups. Median, IQR and range values are presented in pg/ml

TBI test result		N	GFAP, pg/ml			UCH-L1, pg/ml		
	CT		Median	IQR	Range	Median	IQR	Range
TBI test negative	not done	198	16.8	12.2–22.3	2.5–34.2	125.1	80.7-392.6	30.7-392.6
	negative	18	25.5	18.4-31.25	13.2–34.7	241.2	184.4-289.8	59.7-388.3
	positive	0	n/a	n/a	n/a	n/a	n/a	n/a
TBI test positive	not done	82	40.1	21.3–64.4	8.3–1141	359	176.1-535.3	46.8–3846
	negative	150	59.75	37.8-91.15	7.1–1433	479.1	312.9-813.6	45.9–7276
	positive	11	318.7	256-534.7	88.7-735.1	772.1	218.6-942.9	88.5–1275

CT – computed tomography, TBI – traumatic brain injury; N - number of patients; IQR - interquartile range

## Discussion

This study is one of the first to demonstrate the impact of the introduction of the blood TBI test into the routine management of mTBI on patient care in a tertiary German hospital ED. In most hospitals CT scan of the head remains the diagnostic gold standard for mild TBI injury assessment as the only tool able to identify high risk patients with intracranial lesions that might need urgent neurosurgical intervention. However, the percentage of these patients is very small compared to a significant number of suspected mild TBI cases seen in the emergency departments [2, 5]. While guidelines and clinical decision rules exist to standardise selection of patients at higher risk of having intracranial lesions, low compliance is observed among clinicians [3, 6]. Especially in tertiary hospitals with less experienced trainee physicians often on call at the ED, large variations in care and overuse of CT scans have been observed. Recent developments in the field of blood biomarker diagnostics saw the introduction of blood biomarkers to rule out the risk of having intracranial lesions visible on the CT scans [7, 21]. To improve patient care regional trauma centre Klinikum Lüneburg introduced the use of the TBI test into the TBI management protocol for mild TBI patients in the ED. As a tertiary hospital with approximately 600 beds Klinikum Lüneburg does not have a dedicated neurosurgery unit. TBI test was introduced as part of a clinical pathway where it is indicated for all adult patients with GCS 13–15 without high risk factors such as focal neurological deficit, posttraumatic seizures, clinical signs of basal skull fracture, therapeutic anticoagulation, coagulation disorder or a combination of age above 65 and anti-platelet medication. According to the pathway if the test is negative patients can be discharged home without a scan. If the test is positive, either a CT scan or an admission for observation is recommended based on clinical assessment and patient age. Data on the first 460 patients for whom the test was ordered following ED triage is presented in this study. Data showed that the TBI test could rule out 47% of CT scans in mild TBI patients. Some of the patients who could have been ruled out by the test (4%) still had a CT scan ordered based on final clinician’s assessment. None of these patients had any abnormalities on the CT scan, however four of them were subsequently admitted with a TBI diagnosis demonstrating ongoing clinician’s concern. Some patients with a negative (normal) CT scan (43 out of 168) were also admitted for further observation. All admitted patients had a GCS below 15 and displayed clinical symptoms of mild TBI. It has been described that clinical picture does not always correlate with CT scan abnormalities [22]. Moreover, it has been shown in large cohort studies that CT scan abnormalities are not a good predictor of clinical outcomes for mild TBI patients [23, 24]. On the other hand, treating physicians also ruled out additional 12% of CT scans in patients who had a positive test result. These cases show that clinical judgement remains an irreplaceable tool for final assessment and decision-making.

The strength of this study is the presentation of real-world data derived from implementation of the test that was used clinically to rule out a CT scan. This resulted in no imaging outcome data being available for those patients who were ruled out by the test hence it was not possible to calculate diagnostic performance of the test towards CT scan results in the total cohort. However, clinical use data described in this study aligns with numerous previous publications such as multi-centre regulatory trial that demonstrated high safety of the test as a rule-out aid [14], multi-centre studies that looked at performance of GFAP and UCH-L1 biomarkers for CT rule out [25–27] and multiple single-centre studies that evaluated TBI test performance in their specific clinical settings [16, 17, 28–32]. Among 180 patients who had CT scan done, all patients with intracranial lesions (6.6%) had a positive TBI test. ED assessment outcomes also aligned with test results – most patients (88%) with a negative test result were subsequently discharged home. As these patients were not deemed to require any hospital intervention, they represent the portion of suspected TBI patients who benefit most from test introduction, as avoiding an unnecessary CT scan spared them from exposure to a dose of radiation and the extra wait in the ED which would have been required to conduct the scan and receive the radiologist’ report. Exposure to a dose of radiation associated with a head CT scan has been linked to increased risk of cancer, especially of haematological malignancies in children and young people [33, 34]. A recent analysis estimated that current rate of CT scan use could account for 5% of annual cancer diagnoses in the future [35]. Therefore, avoiding unnecessary CT scans improves patient safety.

From hospital operation point of view reducing CT scan orders in the ED improves patient flow and has a potential to positively impact ED waiting times [36, 37]. Blood TBI test use has also been shown to bring cost saving benefits to hospital budgets [38, 39]. Moreover, use of an objective measure such as a blood test result improves clinician’s confidence and patient satisfaction. Large variations in CT use for mTBI patients have been observed across European EDs, therefore introduction of an objective tool such as a blood test has the potential to improve standardisation of the quality of care [3, 4, 36, 37]. This is in line with several guidelines and recent consensus publications that recommend the use of the TBI test, a panel of assays measuring blood concentrations of GFAP and UCH-L1, to rule out the need to conduct a CT scan in patients at low risk of having intracranial lesions. Spanish emergency medicine society recommends the use of the TBI test for all adult mild TBI patients with GCS 13–14 and those with GCS 15 who have risk factors or symptoms [8]. Similarly, French Emergency Medicine Society consensus recommends the use of GFAP and UCH-L1 panel in all adult mild TBI patients seen within 12 h of trauma who are categorized at medium-risk of intracranial lesions [7]. In addition, American College of Surgeons Best Practices Guidelines on the management of traumatic brain injury updated in 2024 also included a recommendation to use TBI biomarkers for all adult mild TBI patients with an indication for a CT scan. Moreover, TBI biomarker measurements are included in the newly proposed Clinical, Biomarker, Imaging and Modifier (CBI-M) framework of characterisation of acute TBI, developed to replace current GCS-based classification to improve and personalise clinical care of TBI patients [40, 41].

This analysis has several limitations. First, data was extracted only for patients who had TBI test done and not from all TBI patients seen within the time period of analysis, therefore it is not possible to report number of patients who did not receive the test due to severity of injury, presence of high-risk factors or time passed since injury. Secondly, time from injury was not extracted from medical records, therefore we cannot evaluate if the 12-hour post-injury window of test use was adhered to in all cases. Thirdly, as the test was used clinically and most patients with a negative result did not have a CT scan, analysis of test diagnostic performance could not be done based on the total cohort. Additionally, pre-implementation data on TBI patient management and CT scan utilisation at Luneburg hospital was not available for comparative analysis, thus the impact of biomarker use on patient management can only be inferred from external literature suggesting that CT scan is being overused in this patient population with up to 95% of scans not detecting any trauma-related abnormalities [5, 6], while criteria for CT scan order are not well standardised among providers [3, 4]. The study is also limited by the lack of follow up data such as return to ED visits and long-term neurological outcomes of the trauma. Finally, due to single-centre study design results could be applicable only to tertiary hospitals and regional trauma centres that adopt a similar clinical pathway of TBI test utilisation. Ready availability of Alinity i^®^ instrument in the Luneburg hospital laboratory and test reimbursement provided in German health system streamlined test adoption at this particular centre, which could be more complex in other centres and health systems that do not have such infrastructure ready for test implementation. The results of this analysis also cannot be extrapolated to specialized trauma centres with neurosurgery units, or out-of-hospital point-of-care use such as field-side, ambulance or community care setting.

This study presents real‑world evidence of a clinical pathway and the impact of adopting routine blood TBI testing, which may inform TBI test implementation in other centres. As more hospital EDs adopt test use following recent mTBI guideline recommendations, and clinicians develop understanding and experience with test results, more data will become available to further evaluate its impact on clinical practice. Future multicentre studies analysing data coming from test implementation across different health systems will provide health system-specific validation of test adoption impact on patient care and on the ED and radiology services utilisation. In the next stages of test adoption it could have additional clinical utility as research studies are showing that TBI biomarkers could be useful in evaluation of patients seen after the first 12 h [42], those with moderate and severe TBI [43], in paediatric patients [44], and patients with other neurological conditions [45, 46].

## Conclusions

This retrospective analysis of real-world clinical adoption of the blood TBI test showed that the newly introduced test is safe and effective for CT scan rule out. Adoption of the clinical pathway incorporating the blood test allowed this single ED to safely discharge over 40% of suspected mTBI patients without a scan being conducted. Such advancement in mTBI management elevates patient care by limiting exposure to radiation and providing tools for standardised care optimised to minimise waiting time in the ED and demand for radiology services.

## Data Availability

The raw data supporting the conclusions of this article will be made available by the authors upon reasonable request.

## Abbreviations

mTBI: Mild Traumatic Brain Injury
CT: Computed Tomography
NPV: Negative Predictive Value
ED: Emergency Department
