# Correction: Impact of routine use of the GFAP and UCH-L1 TBI blood test for management of mild TBI on patient care in a European emergency department

**DOI:** 10.1007/s00068-026-03336-9

**Published:** 2026-09-07

**Authors:** Felix Brüning-Wolter, Nicola Wolff, Meike Schrader, Ksenia Musaelyan, Raj Chandran, Markus Hoffmann, Jaime A. Marino, Beth McQuiston, Jörg Cramer

**Affiliations:** 1 Department of Trauma Surgery and Orthopaedics, Marienkrankenhaus, Hamburg Germany; 2 Emergency Department, Städtisches Klinikum Lüneburg, Lüneburg, Germany; 3 Laboratory Medicine Department, Städtisches Klinikum Lüneburg, Lüneburg, Germany; 4 Abbott Core Diagnostics, Abbott Park, IL USA; 5 Orthopedics and Traumatology Department, Städtisches Klinikum Lüneburg, Lüneburg, Germany

**Correction: European Journal of Trauma and Emergency Surgery (2026) 52:251**

**https://doi.org/10.1007/s00068-026-03303-4**

The corresponding author is not indicated correctly in the online article. The first author, Dr Felix Bruning-Wolter, should be indicated as the corresponding author. While his correct email address is listed in the PDF title page, the name of the corresponding author is not indicated correctly.

Incorrect version:

Jörg Cramer

bruewo@gmail.com

Correct version:

Felix Brüning-Wolter

bruewo@gmail.com

The Original Article has been corrected.

